# Evaluation of Human Bone Marrow Mesenchymal Stromal Cell (MSC) Functions on a Biomorphic Rattan-Wood-Derived Scaffold: A Comparison between Cultured and Uncultured MSCs

**DOI:** 10.3390/bioengineering9010001

**Published:** 2021-12-21

**Authors:** Payal Ganguly, Jehan J. El-Jawhari, James Vun, Peter V. Giannoudis, Elena A. Jones

**Affiliations:** 1 Leeds Institute of Rheumatic and Musculoskeletal Medicine, University of Leeds, Leeds LS9 7TF, UK; P.Ganguly@leeds.ac.uk (P.G.); j.vun@nhs.net (J.V.); p.giannoudis@leeds.ac.uk (P.V.G.); 2 Department of Biosciences, School of Science and Technology, Nottingham Trent University, Nottingham NG11 8NF, UK; jehan.el-jawhari@ntu.ac.uk; 3 Department of Clinical Pathology, Mansoura University, Mansoura 35516, Egypt; 4 Leeds Orthopaedic & Trauma Sciences, Leeds General Infirmary, University of Leeds, Leeds LS9 7TF, UK; 5 Leeds Musculoskeletal Biomedical Research Centre, Chapel Allerton Hospital, Leeds LS7 4SA, UK

**Keywords:** mesenchymal stromal cells (MSCs), biomorphic scaffolds, bone regeneration, gene expression

## Abstract

The reconstruction of large bone defects requires the use of biocompatible osteoconductive scaffolds. These scaffolds are often loaded with the patient’s own bone marrow (BM) cells to facilitate osteoinductivity and biological potency. Scaffolds that are naturally sourced and fabricated through biomorphic transitions of rattan wood (B-HA scaffolds) offer a unique advantage of higher mechanical strength and bioactivity. In this study, we investigated the ability of a biomorphic B-HA scaffold (B-HA) to support the attachment, survival and gene expression profile of human uncultured BM-derived mesenchymal stromal cells (BMSCs, *n* = 6) and culture expanded MSCs (cMSCs, *n* = 7) in comparison to a sintered, porous HA scaffold (S-HA). B-HA scaffolds supported BMSC attachment (average 98%) and their survival up to 4 weeks in culture. Flow cytometry confirmed the phenotype of cMSCs on the scaffolds. Gene expression indicated clear segregation between cMSCs and BMSCs with MSC osteogenesis- and adipogenesis-related genes including *RUNX2, PPARγ, ALP* and *FABP4* being higher expressed in BMSCs. These data indicated a unique transcriptional signature of BMSCs that was distinct from that of cMSCs regardless of the type of scaffold or time in culture. There was no statistical difference in the expression of osteogenic genes in BMSCs or cMSCs in B-HA compared to S-HA. VEGF release from cMSCs co-cultured with human endothelial cells (*n* = 4) on B-HA scaffolds suggested significantly higher supernatant concentration with endothelial cells on day 14. This indicated a potential mechanism for providing vasculature to the repair area when such scaffolds are used for treating large bone defects.

## 1. Introduction

Bone healing and repair following trauma remains a challenging physiological process, especially for patients who suffer from severe fracture injuries or have associated comorbidities such as obesity or diabetes. The risk of an impaired healing response is also higher in patients with osteoarthritis, who may need surgical treatments alongside drug intervention for enhanced osseointegration of the implants [1,2]. This impaired bone healing process often leads to a greater risk of fracture non-union, a complication that could require multiple surgical interventions with increased medical and societal costs [3].

Lately, the use of biomaterial scaffolds using the so-called ‘diamond concept’ has significantly improved the bone healing response and outcomes in such difficult clinical situations [4]. In brief, the concept involves the use of a platform/biomaterial that serves as the scaffold (matrix) onto which bone forming cells are added or can migrate to, under the influence of growth factors (inductive molecules) to encourage bone regeneration. Finally, the mechanical strength of the metalwork, as well as the scaffold itself, plays a crucial role in providing the necessary stability to the repair area.

Bone by itself is an extremely dynamic organ with unique microarchitecture to support the formation and activities of a plethora of cells inside its niche. Within the bone, the bone marrow (BM) compartment is the seat of these activities that determine the ability of the bone to repair, regenerate and become healthy once again. The progenitors of bone residing within the BM are mesenchymal stromal cells (MSCs) that drive the process of osteogenesis with the help of various growth factors [5]. MSCs provide niche support to haematopoietic and endothelial cells; differentiate into at least bone, fat and cartilage and are increasingly being used for the investigation of bone regeneration processes in vitro, including those on different types of biomaterial scaffolds [6]. 

Interestingly, the majority of the studies performing these investigations are utilising culture-expanded MSCs (cMSCs) [7,8,9]. Nonetheless, using cMSCs does not necessarily mirror the bone healing mechanisms in vivo as they themselves undergo replicative senescence during culturing over time [10]. On the other hand, MSCs from fresh BM aspirates withdrawn from patients (BMSCs) are increasingly used in the orthopaedics practice as a one-stage surgical procedure, either alone or following loading on a relevant bone scaffold [11]. Although there exist a plethora of studies investigating the behaviour of cMSCs on biomaterial scaffolds, similar studies with BMSCs remain scarce but provide a better insight towards repair mechanisms that occur following scaffold implantation.

Large 3D porous hydroxyapatite scaffolds with biorelevant ionic substitutions (B-HA scaffolds) have been recently produced through the biomorphic transformation of a natural wood (rattan wood pieces or *Calamus manna*) [12,13]. Compared to sintered, HA-based ceramics with isotropic porosity, B-HA scaffolds contain hierarchically organised, wide, aligned channels of ~300 μm and smaller connected tubules mimicking the microstructure and the mechanical stiffness of the cortical bone shell, thus, making them ideally suited for weight-bearing, long-bone fracture repair. The scaffolds are prepared using various manufacturing steps to encourage the dissolution of calcite and re-precipitation of HA-nanocrystals of ~20 nm in thickness and ~150 nm in width and are additionally reinforced with Mg^2+^ and Sr^2+^ ions for enhanced biocompatibility, osteogenesis as well as better mechanical properties [14]. Previous biocompatibility studies using B-HA scaffolds have been performed using cultured BM MSCs from mice (C57BL/6) [12] or human adipose tissue–derived stem cells [14]. No study as yet has investigated their support for human BM MSCs, particularly those from unprocessed BM aspirates (BMAs). 

The principal aim of this study was, therefore, to evaluate the support functions of B-HA scaffolds towards rare, uncultured BMSCs from human BMAs and compare BMSCs with cMSCs. Our secondary aim was to compare B-HA scaffolds with a sintered HA scaffold (S-HA) with a classical macro-, micro- and interconnected porosity. As a result, we provide evidence for MSC attachment, survival and gene expression of transcripts associated with multiple MSC functions including bone formation and angiogenesis support on B-HA and demonstrate their release of VEGF protein essential for vascular formation around the construct. 

## 2. Materials and Methods

### 2.1. Ethics and Sample Processing

Ethical approval was obtained from the NREC Yorkshire and Humberside National Research Ethics Committee (numbers 18/YH/0166 and 06/Q1206/127) for the collection of samples from human donors.

For seeding the scaffolds with cMSCs, frozen vials of previously culture-expanded and characterised passage 2 MSCs (*n* = 7 donors, age range 19–65 years old) were defrosted in complete DMEM media containing 10% FBS and 1% P/S (Thermo Scientific, Loughborough, UK) and cultured in StemMACS (Miltenyi Biotec, Bisley, UK) (SM) to obtain up to 2 × 10^6^ MSCs.

For seeding the scaffolds with fresh BM aspirates (BMAs), BMA was collected from patients undergoing metal removal or fracture reconstruction (*n* = 6 donors, age range 36–65 years old). Three additional donors (age range 41–52) were recruited for VEGF ELISA measurements. The patients were deemed to be otherwise healthy. An average of 4 mL of BMA was collected in tubes containing ethylenediamine tetraacetic acid (EDTA) to prevent clotting. The collected tubes were then taken to the laboratory for further processing.

**Figure 1:**
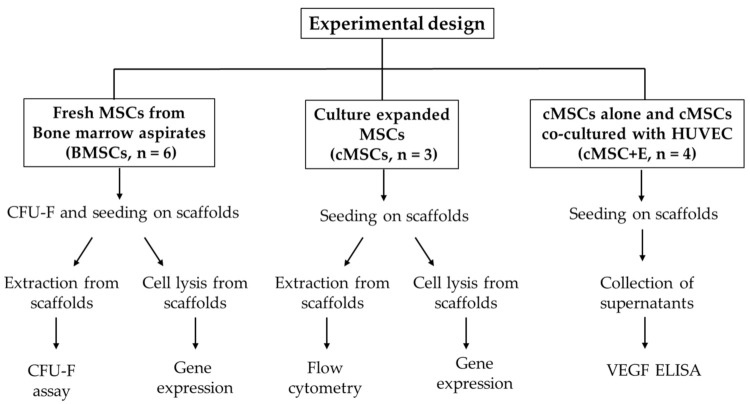
Schematic summary of all the experiments performed.

A simple schematic summary of all the experiments performed is shown below in Figure 1.

### 2.2. Scaffolds, Cell Loading and Culturing on Scaffolds

B-HA manufacture and physical and mechanical properties have been described previously [12]. In brief, cylindrical rattan wood pieces (*Calamus manna*) were transformed to CaCO_3_ using a series of physicochemical treatments and gas under supercritical conditions [12]. Then, the obtained CaCO_3_ body was finally transformed through hydrothermal reactions into a biomorphic apatite body presenting multiple doping with Mg^2+^ and Sr^2+^. The resulting scaffolds were 10 mm in diameter with a specific surface area of 12.30 m^2^ g^−1^ [12]. Commercially available, porous HA scaffolds (S-HA) with an almost 90% porosity and broadly similar size and surface area (diameter 9 mm, surface area of 64 mm^2^) and main pore size of 100–200 μm were used as controls. Microscopic images (AMG EVOS fl-fluorescence microscope) of both scaffolds at 4X using transmitted light have been provided in Supplementary Figure S1 to highlight the scaffold structures. Before adding cMSCs or BMSCs for in vitro biological evaluations, the scaffolds were primed in SM at 37 °C and 5% CO_2_ for 72 h. This was performed to enable the ionic equilibrium in the media [12] around the scaffolds, as well as to provide MSC attachment factors in the SM media [15]. For loading scaffolds with BMA, primed B-HA scaffolds were placed in a 24-well plate and loaded with 316 μL of BMA (in duplicate) for 3 h at 37 °C. To account for small differences in scaffold volumes, duplicate S-HA scaffolds were loaded with 256 μL of BMA.

To measure rare BMSC attachment to the scaffolds, CFU-F assay was used as previously described [15]. For this, 200 μL BMA was seeded in duplicate 100 mm Petri dishes (Corning, New York, NY, USA) containing 10 mL SM media as ‘before attachment’ controls and cultured for 2 weeks as per the standard colony forming unit fibroblast (CFU-F) assay protocol [16,17]. After the 3 h incubation period of BMA with the scaffolds, the latter was moved to low-attachment 24-well plates and covered with 1.5 mL SM media. After 24 h, a complete media change was performed, and the aspirated media (containing any non-attached MSCs) were plated in another set of duplicate CFU-F dishes to ultimately calculate the proportions of non-attached MSCs. The attached MSCs were subsequently calculated by subtraction [15]. To study long-term MSC survival in standard, static conditions, the scaffolds were cultured in low-attachment 24-well plates (Scientific Laboratory Supplies, Nottingham, UK) in SM media for 1, 2 and 4 weeks with half-media changes performed twice a week. 

For seeding the scaffolds with cMSCs, the frozen vials of passage 2 cells were defrosted and revived in SM media overnight before seeding them on the scaffolds. On the day of seeding, 2 × 10^5^ MSCs per scaffold were re-suspended in 316 μL of SM media and added to primed B-HA scaffolds in duplicate for 3 h. Similarly, 1.6 × 10^5^ cells in 256 μL were added to S-HA scaffolds owing to their smaller surface area. After the incubation, the scaffolds were moved to a low-attachment 24-well plate and cultured for 1, 2 and 4 weeks. Similarly to the cultures established with BMSCs, each well with a cMSC-seeded scaffold was fed with 1.5 mL of SM media and a half-media change was performed twice weekly. The attachment of cMSCs was calculated by counting any non-attached cells after 24 h and relating it to the numbers of seeded cMSCs.

### 2.3. Cell Extraction from Scaffolds to Test for MSC Survival

The extraction of cells from the scaffolds was performed from one of the duplicate scaffolds following the collagenase/trypsin treatment protocol, as previously described [15]. At the end of each time point, scaffolds were washed three times with 2 mL PBS and first treated with 0.5 mL trypsin (both from Life Technologies, Paisley, UK) for 10 min in 37 °C. The activity of trypsin was blocked by the addition of 10 µL of volume for 10% FCS for 5 min. Next, 0.5 mL of collagenase (Stem Cell Technologies, Vancouver, BC, Canada) was added to the scaffolds and incubated at 37 °C for 0.5 h with regular manual agitation to ensure the removal of all cells. The collagenase fraction was then added to the trypsin fraction and passed through a 70 μm cell strainer (Thermo Fisher Scientific) to remove any debris from the scaffolds. The released cells were counted using trypan blue and used for subsequent flow cytometry (for cMSCs, see below section) or CFU-F assay (for BMSCs) for evaluation of MSC maintenance. For CFU-F assay with cells released for the scaffolds loaded with BMAs, the released cells were seeded into six-well plates (Corning). Any colonies derived from released cells were counted after staining with methylene blue and compared to seeded MSCs.

### 2.4. Flow Cytometry

To prove the MSC identity of the cells released from the scaffolds seeded with cMSCs, flow cytometry was performed as previously described [18]. Released cells were distributed in two FACS tubes and stained with CD45 Viogreen and CD90-FITC (tube 1) or CD105 FITC and CD73 PE (tube 2) (all reagents from Miltenyi Biotec) for 15 minutes in the dark before washing and centrifuging to obtain a pellet. The pellets were then re-suspended in 500 μL FACS buffer containing 7-AAD (Miltenyi Biotec) and analysed using a flow cytometer, Attune (Thermo Fisher Scientific, Waltham, MA, USA). All the events were displayed on forward (FSC) and side scatter (SSC) images. Following this, doublets and any other events were eliminated to gate cells only using the height and area of FSC (FSC-A and FSC-H). After that, 7-AAD was used to identify the live cells from which MSCs were evaluated using the expression of CD105, CD73 and CD90 and the lack of CD45.

### 2.5. Gene Expression

The second scaffolds’ duplicate was treated with 350 μL of Buffer RL (Norgen Biotek, Thorold, ON, Canada) to lyse the cells inside the scaffolds with strong agitation using vortex over ice to ensure removal of all the cells with their genetic contents and prevent RNA degradation. Gene expression was performed on a predetermined panel of genes associated with MSC functionalities (Supplementary Table S1) as per methods described previously [19,20]. Briefly, RNA was extracted from the cells from the scaffolds collected in the buffer RL and reverse transcribed to complementary DNA (cDNA). The cDNA was pre-amplified using 14 cycles and PA master mix using Fluidigm (Fluidigm, San Fransisco, CA, USA) reagents as per the manufacturer’s protocols for scaffolds established from cultured MSCs or BMAs, respectively. Quantitative polymerise chain reaction (qPCR) was then performed on a Fluidigm 48.48 Dynamic Array^TM^ integrated fluid circuit (IFC, Fluidigm) using *HPRT1* and as the housekeeping gene. The Ct values of the genes of interest were normalised to the housekeeping gene and then calculated for their relative expression as 2^−^^Δ^^Ct^ using the formula ΔCt = Ct_target gene_ − Ct_housekeeping gene_. Cluster (version 3.0) and Java TreeView (version 1.1.6r4) software were used to generate clusters of gene expression of log2 transformed data for visualisation via heatmaps. The expression of genes is indicated in red (+3, highest) or green (−3, lowest) compared to *HPRT1*. Grey squares indicate values that were below detection levels, and black squares indicate values close to 1. 

### 2.6. Enzyme Linked Immunosorbent Assay (ELISA)

ELISA was performed to measure VEGF in supernatants collected from B-HA/cMSCs’ constructs (*n* = 4) seeded either alone or co-cultured along with human umbilical vein endothelial cells (Promocell, Heidelberg, Germany) for 2 weeks. HUVECs were grown to confluence in T75 flasks using endothelial media (Promocell) and seeded at 1 × 10^5^ HUVECs per scaffold in duplicate, together with 1 × 10^5^ cMSCs per scaffold in media containing endothelial media: SM media in the ratio 1:1 to ensure growth of both the cMSCs as well as the HUVECs. Scaffolds seeded with MSC only (2 × 10^5^ cMSCs per scaffold) served as controls, and the supernatants were collected on days 1, 7 and 14 and frozen at −80 °C. A full-media change was performed on day 1 followed by half-media changes twice/week. The ELISA was performed as per the manufacturer’s protocol. In brief, supernatant samples were defrosted, and the VEGF standard dilutions were prepared and added to the 96-well plate. Next, 50 μL of sample diluent followed by 50 μL of supernatant sample was added to the remaining wells in a pre-determined sequence. The plate was incubated for 2 h at room temperature (RT) followed by the addition of biotin conjugate, streptavidin–horseradish peroxidase (HRP), 3,3′,5,5′-tetramethylbenzidine (TMB) solution and stop solution with washing and incubation in between each step. Finally, the colour change of the plate (VEGF ELISA Kit, Invitrogen) was read at 450 nm using the Cytation 5 imaging plate reader (Bio Tek), and GraphPad Prism software (version 9.2) was used to produce a standard curve and generate values of VEGF for all samples.

### 2.7. Statistics

Statistical analysis and graphical representations of the data were performed using GraphPad Prism (version 9.2.0). Normal distribution of the data was checked using the Shapiro–Wilk and Kolmogorov–Smirnov tests. For data that were not found to be normally distributed, the Mann–Whitney test was used for unpaired data and the Wilcoxon signed rank test for paired data. Statistical analysis of data across three time points (week 1, week 2 and week 4) was carried out using one-way ANOVA followed by Kruskal–Wallis non-parametric test with Dunn correction for multiple groups. The results were considered significant at a *p* value of <0.05.

## 3. Results

### 3.1. cMSCs and BMSC Attachment and Survival on B-HA Scaffolds

**Figure 2:**
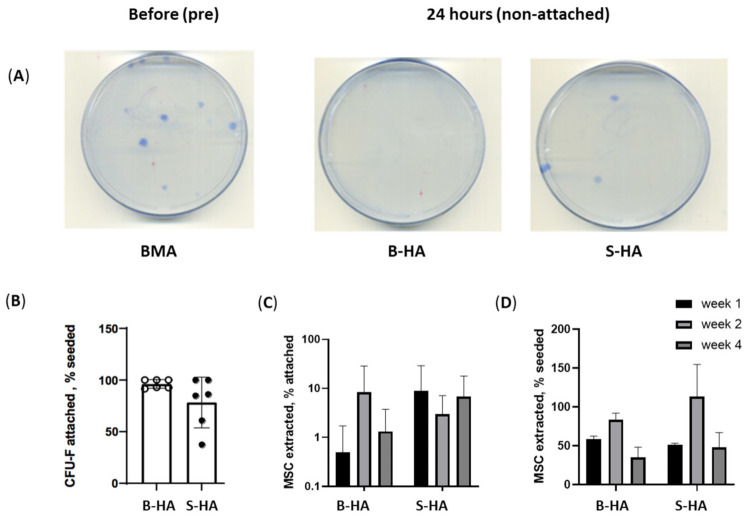
BMSC and cMSC attachment and survival on the scaffolds. (**A**) Examples of BMSC colonies (CFU-F) following the plating of BMA without any scaffolds (**left**), BMSC colonies/CFU-F non-attached on B-HA (**centre**) and S-HA (**right**) after 24 h of adding BMA to the scaffolds. (**B**) BMSC attachment to scaffolds measured as the percentage of attached CFU-Fs to both scaffolds relative to seeded CFU-F; (**C**) BMSC maintenance measured as the percentage of BMSCs extracted after week 1, 2 and 4 of culture; (**D**) cMSC maintenance measured as the percentage of cMSCs extracted after week 1, 2 and 4 of culture using trypan blue for cell counts. Data are shown with mean and standard error of mean and with *n* = 3 cMSCs and *n* = 6 BMSCs.

The aim of these experiments was to investigate and compare the attachment of cMSCs and BMSCs to B-HA scaffolds and to evaluate their survival in comparison to S-HA scaffolds during long-term culture in static conditions. To study BMSC survival, BMA-loaded scaffolds were maintained in culture for 4 weeks. Cells were extracted from the scaffolds at week 1, 2 and 4, and BMSCs were quantified using a CFU-F assay (Figure 2).

To analyse the attachment of fresh BMSCs to the scaffolds, unprocessed BMA was added to the scaffolds for 3 h, after which the scaffolds were transferred to a low-attachment 24-well plate and maintained in culture. The same unprocessed BMA was plated on 100 mm Petri dishes in the CFU-F assay as ‘before attachment’ control (Figure 2A(left)). After 24 h, full-media change was performed, and all BMA cells non-attached to the scaffolds were plated in 100 mm Petri dishes to establish the proportion of unattached BMSCs (Figure 2A(right)), from which the proportion of attached BMSCs was calculated by subtraction. The analysis revealed that B-HA scaffolds provided better BMSC attachment than S-HA; however, the differences were not statistically significant (Figure 2B(left)). 

The relatively lower percentage of cells extracted from the B-HA scaffolds could be attributed to their unique longitudinal porosity, which is a characteristic of the scaffold, making it difficult to extract cells that were trapped within these distinctive pores. This is observed especially at week 1, when the resorption rate is the lowest (Figure 2C,D). It is speculated that, with the passage of time in weeks, the rate of resorption increased, and it was easier to extract the cells from the nanostructural pores indicated.

Finally, scaffolds’ support to uncultured BMSCs was compared to their support for cMSCs. Following seeding, BMSCs and cMSCs were extracted at week 1, 2 and 4, and results indicated no significant differences between the scaffolds and a higher BMSC and cMSC survival at week 2 (Figure 2C,D). Altogether, these data indicated that both scaffolds supported the attachment and survival of cMSCs at least up to week 2 in standard, static conditions.

**Figure 3:**
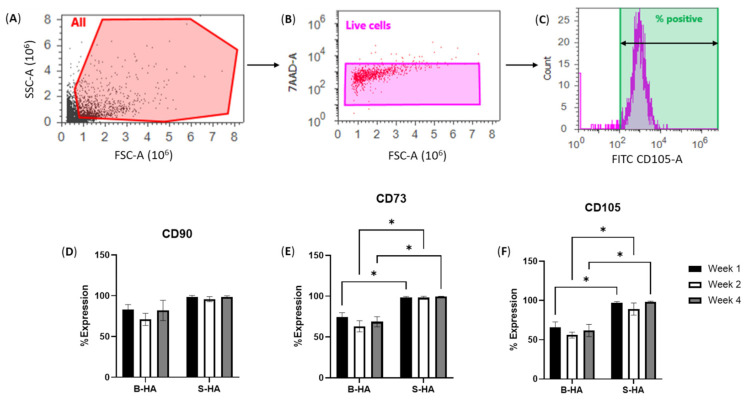
Phenotypic analysis of cMSCs after extraction from B-HA and S-HA scaffolds. (**A**–**C**) Gating strategy from all events (**A**) to gating on live 7-AAD-negative cells (**B**) and calculation the percentage of marker-positive cells based on histogram (**C**). (**D**–**F**) Percentage expression of MSC markers on the extracted cells from both scaffolds at week 1, week 2 and week 4 for CD90 (**D**), CD73 (**E**) and CD105 (**F**). Results are shown as mean with standard error of mean for *n* = 3 cMSCs. * *p* < 0.05.

To confirm the MSC identity of the cells extracted from the scaffolds, they were analysed using flow cytometry using a panel of standard MSC markers [21] (gating strategy described in Figure 3A–C). The data were analysed as a percentage of marker-positive cells relative to total live cells (Figure 3). A higher percentage of CD73-, CD105- and CD90-marker-positive cells were observed from the cells extracted from S-HA. These differences were statistically significant as compared to those extracted from B-HA scaffolds for CD105 at week 1 (*p* = 0.0404), week 2 (*p* = 0.0221) and week 4 (*p* = 0.0406) and also for CD73 at week 1 (*p* = 0.0329), week 2 (*p* = 0.0283) and week 4 (*p* = 0.0388). The decline in MSC markers in cells seeded on B-HA scaffolds indicated their potential differentiation, which was studied next using gene expression evaluation.

### 3.2. Gene Expression of cMSCs and BMSCs Extracted from Scaffolds

Gene expression analysis of 48 transcripts (Supplementary Table S1) was performed on cMSCs and BMSCs lysed from both scaffolds at week 1, 2 and 4 of culture. 

**Figure 4:**
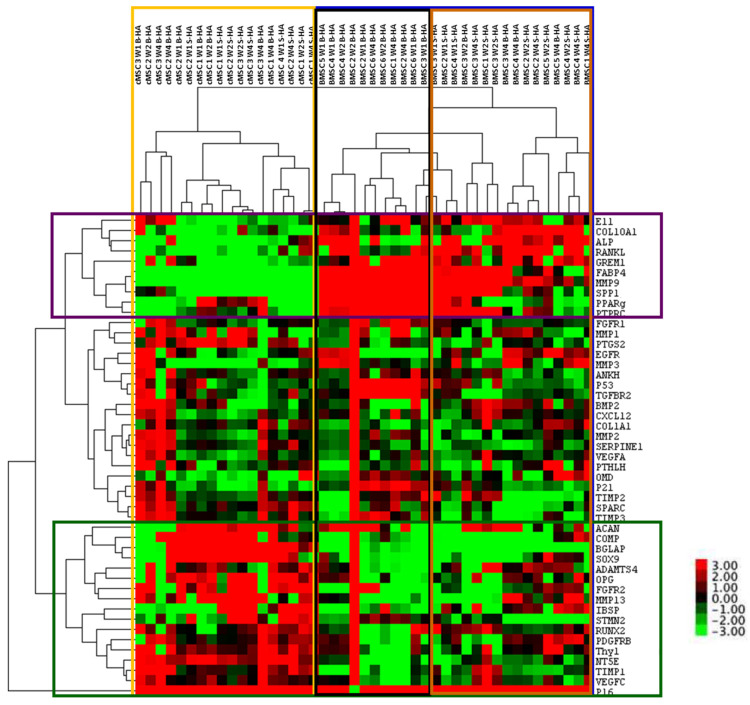
Cluster analysis of all samples and all genes. Cluster analysis of cMSCs and BMSCs after lysis from B-HA and S-HA scaffolds. Labels on the right indicate the genes, and labels on the top indicate samples. The range of gene expression levels varies from the highest expression in red to the lowest in green. Their colour key for the relative expression is on the right. Data are presented for *n* = 3 cMSCs and *n* = 6 BMSCs, coloured boxes are explained in the text.

The cluster analysis revealed clear segregation of BMSCs, outlined in a blue box on the right, and cMSCs, outlined in a yellow box on the left, irrespective of the time in culture and the scaffolds used (Figure 4). For BMSCs, a further two clusters could be observed, outlined by black and brown boxes, the former being enriched for B-HA samples and the latter being enriched for S-HA samples. This segregation was not as obvious in cMSC samples, suggesting greater differences between the scaffolds when they are loaded with uncultured BMSCs. 

In terms of gene clustering, three major groups of genes were evident: those with an overall higher expression in BMSCs (top cluster, genes outlined in purple), those with an overall higher expression in cMSCs (bottom cluster, genes outlined in green) and those with similar expression (middle cluster, genes in between the purple and green clusters). For example, osteogenesis- and bone-formation-related E11, COL10A1, ALP, RANKL, OPN and adipogenesis-related PPARγ and FABP4 were highly expressed in BMSCs as compared to cMSCs (outlined in purple). This potentially suggests that BMSCs on B-HA maintained higher osteogenic and adipogenic abilities compared to cMSCs. Interestingly, the chondrogenic transcripts ACAN, COMP and SOX9 were overall higher expressed in cMSCs than in BMSCs (outlined in green), potentially suggesting a better chondrogenic ability for cMSCs than for BMSCs when cultured on these scaffolds. 

### 3.3. Gene Expression Changes in cMSCs Present in Both Scaffolds

**Figure 5:**
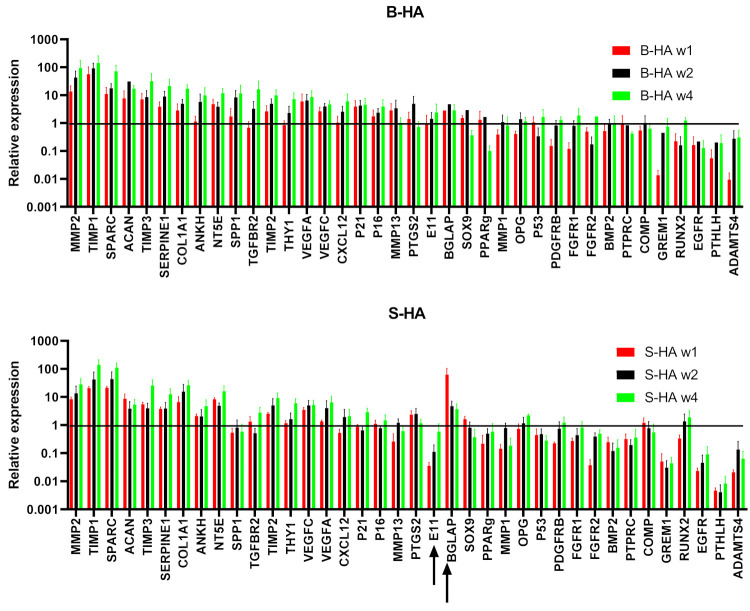
Gene expression of cMSCs normalised to HPRT. Top panel shows the gene expression of transcripts on B-HA, and the bottom panel shows gene expression of transcripts on S-HA for week 1, 2 and 4. Data were normalised to the housekeeping gene *HPRT1*, indicated with the black horizontal line. Black arrows indicate genes that displayed expression levels lower than that of the housekeeping gene. Data are shown as mean with standard error of mean for *n* = 3 cMSCs.

**Figure 6:**
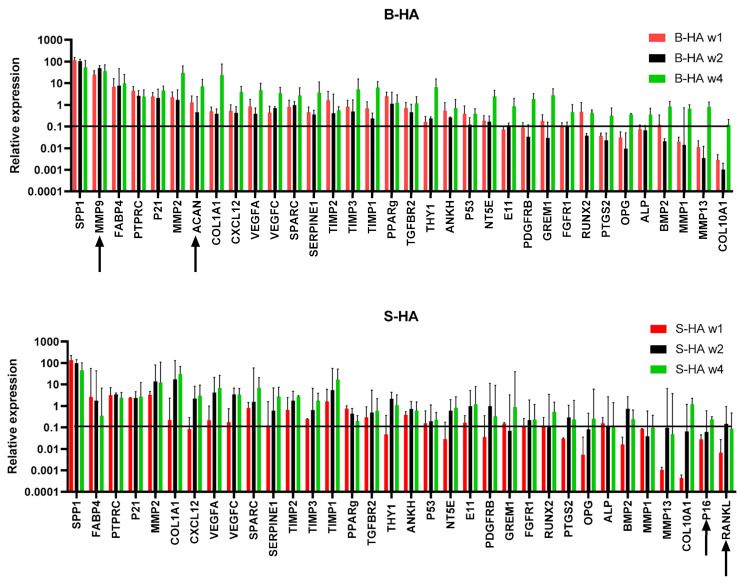
Gene expression of BMSCs normalised to HPRT. Top panel shows the gene expression of transcripts on B-HA, and the bottom panel shows gene expression of transcripts on S-HA for week 1, 2 and 4. Data were normalised to the housekeeping gene *HPRT1*, indicated with the black horizontal line. Black arrows indicate genes that displayed expression levels lower than that of the housekeeping gene. Data are shown as mean with standard error of mean for *n* = 6 BMSCs.

As the cluster analysis revealed clear segregation between cMSCS and BMSCs (Figure 4), a more detailed analysis of their gene expression was next performed on these cells separately (Figure 5 and Figure 6). It was found that 38 out of 48 transcripts (79%) were highly and consistently expressed in cMSCs present in B-HA and S-HA scaffolds, and these are shown in Figure 4. As seen from this figure, all cMSCs expressed high levels of transcripts encoding many proteins and enzymes involved in extracellular matrix (ECM) turnover (including different MMPs and TIMPs) as well as several bone- and cartilage-specific structural proteins such as SPARC and ACAN. The cells also expressed classical MSC transcripts NT5A and THY1; trophic factors VEGFA, VEGFC and CXCL12 and all three MSC lineage transcription factors (TFs): Sox9 (cartilage), PPARγ (fat) and RUNX2 (bone). This gene expression pattern was consistent with cMSCs, which are characterised by a notable absence of mature, fat-lineage proteins [22]. There were no notable differences between cMSCs present in B-HA and S-HA other than a less uniform expression of E11 (a marker of early osteocytes) and BGLAP in S-HA (Figure 5). In addition, there were no statistically significant increases or decreases in gene expression levels with time in culture, although a number of the transcripts displayed a trend for increased levels from week 1 to week 4, potentially indicating higher transcriptional activity in the cells at week 4. 

### 3.4. Gene Expression of BMSCs Present in Both Scaffolds

In all, 34 out of 48 transcripts (71%) were highly and consistently expressed in BMSCs present in B-HA and S-HA scaffolds (Figure 6). Similar to scaffolds seeded with cMSCs, BMSCs expressed high levels of transcripts encoding ECM proteins and enzymes as well as several bone-specific structural proteins such as SPARC and SPP1. In addition to classical MSC transcripts NT5A and THY1, a relatively high expression of PTPRC indicated the attachment and persistence of haematopoietic-lineage cells from BMA, some of which (such as macrophages) could be useful for bone regeneration [23,24]. BMSCs also expressed relatively high levels of VEGFA, VEGFC and CXCL12 as well as bone- and fat-lineage TFs RUNX2 and PPARγ. Compared to cMSCs, the cells displayed prominent expression of fat-lineage mature protein FABP4 and lacked Sox9 TF expression, indicating a tilt towards adipogenesis, characteristic for BMSCs compared to cMSCs [22,25]. There were some differences between B-HA and S-HA scaffolds, such as notable expression of ACAN and MMP9 in BMSCs present in B-HA and higher/more consistent expression of p16 (senescence marker) and RANKL (bone resorption driver) in BMSCs present in S-HA (Figure 6). Similar to cMSCs, there were no statistically significant increases or decreases in gene expression levels with time in culture, although the majority of the transcripts displayed a trend for increased levels from week 1 to week 4, potentially indicating higher transcriptional activity in the cells at week 4. 

### 3.5. VEGF Production by cMSCs Alone or in Co-Culture with Endothelial Cells on B-HA Scaffolds 

**Figure 7:**
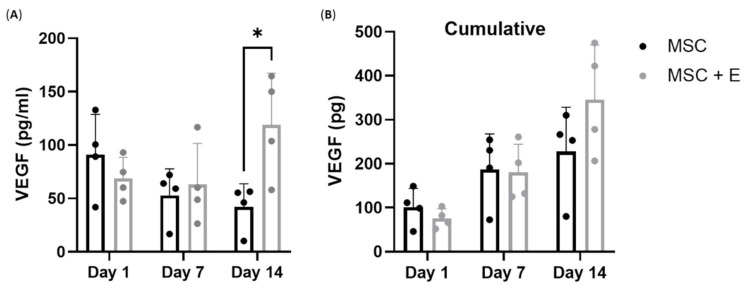
VEGF production by cMSCs cultured on B-HA. (**A**) VEGF concentrations in supernatants collected from cMSCs in comparison with cMSCs co-cultured with endothelial cells (+E) in B-HA and (**B**) cumulative VEGF release with time in culture. Results are shown as mean with standard error of mean for *n* = 4 cMSCs. * *p* < 0.05.

VEGF production by MSCs is critically important for bone regeneration [26,27]. As we detected VEGF expression in cMSCs at the transcript level, it was important to evaluate VEGF protein release by MSCs cultured on B-HA, which we next measured at days 1, 7 and 14 of culture using ELISA (Figure 7A). Over the 14 days, a half-media change was performed to maintain the culture. This would impact the level of VEGF produced. For example, on day 1, the level would be high, and after the following media change, the concentration of VEGF would be reduced. Thus, to account for the media change affecting the VEGF concentrations, data were also presented as cumulative VEGF levels (Figure 7B).

The concentration of VEGF in all samples on day 1 was between 50 and 150 pg/mL (Figure 7A). On day 1, the VEGF level was higher in supernatants from cMSCs than in cMSCs + E, as expected [28]. From day 7 onwards, the supernatant levels of VEGF declined in cMSCs but not in cMSCs + E cultures. As the cultures were fed with fresh media several times during these two-week-long experiments, VEGF cumulative release was calculated, taking media dilutions into account, and revealed an increase in VEGF production, particularly in cMSCs + E cultures (*p* = 0.0129), potentially indicating a paracrine mechanism of its production. These data confirmed the viability and functional angiogenesis-supporting potency of cMSCs present in B-HA scaffolds. 

For comparison with cMSCs, we also investigated the VEGF levels from BMSCs from *n* = 3 donors in standard 2D (without scaffolds) conditions. We found the VEGF release to be between 99 and 148.7 pg/mL on day 14 from an average of 10^4^ seeded BMSCs (Supplementary Table S2). 

## 4. Discussion

Fracture non-union and bone defects, particularly in weight-bearing long bones, continue to represent a significant clinical challenge. Firstly, the natural architecture of a long bone, as well as the need to withstand considerable and directional mechanical loads, dictates the development of scaffolds with very high mechanical strength, anisotropy, complex geometry and multiscale porosity [29,30]. Secondly, bone reconstruction with these synthetic scaffolds requires biological stimulation, either in a form of growth factors or osteogenic stem cells such as autologous cMSCs or BMSCs. The latter are preferentially used by the orthopaedic surgeons as they are not regulated as advanced therapy medicinal products (ATMPs) [31]. Finally, blood supply to the entire repair area remains a considerable challenge and requires novel approaches in scaffold fabrication, specifically for angiogenesis stimulation [13,29]. Biomimicking is a new development in bone scaffold fabrication, which capitalises on the evolutionally developed, highly hierarchical structures that serve as sacrificial templates for chemical transformation and reinforcement [32]. Wood in particular has a highly anisotropic structure characterised by long parallel channels (used in plants to transport water and nutrients) that have variable lumen diameters and inherent interconnectivity [32] that closely mimic the Haversian and Volkmann canals in human cortical bone [33]. Rattan wood has another advantage of having evenly distributed directional channels (with no seasonal rings) [34], which are particularly suitable to act as conduits for blood vessel ingrowth, as well as the lacunar fractal nature of porosity, influencing its material strength and stiffness [34]. 

Previous studies have investigated the biomechanical properties and biocompatibility of rattan-templated biomorphic scaffolds (B-HAs) using primary mouse and human cells, including adipose-derived stem cells (ASCs) [12,13,14,30,34]. The current study is the first to investigate the attachment, survival and gene expression of MSCs harvested from human bone marrow, the MSC source easily accessible to an orthopaedic surgeon and commonly used for bone reconstruction, with a particular emphasis on uncultured BMSCs that do not require ATMP regulation. BM as an easily accessible source of BMSCs was used in this study to closely reflect in vivo conditions. Towards the third challenge in large-scale defect reconstruction, this study investigated the long-term release of VEGF following B-HA scaffold colonisation by cMSCs. 

Previously, Sprio et al. have demonstrated the biocompatibility of B-HA scaffolds using mouse (C57BL/6) MSCs and human adipose-derived stem cells (hADSCs). In their first study, they demonstrated the use of Sr^2+^-substituted scaffolds for enhanced bone formation abilities, bioactivity and biocompatibility in a rabbit animal model [35]. In another study, they further confirmed their enhanced bioactivity and compressive strength using human-derived ADSCs [13]. In contrast, the current study focused on patient-derived uncultured BMSCs, as these cells most closely reflect the bone marrow in vivo. Furthermore, their initial work used dynamic seeding, and their most recent work explored the gene expression of osteogenic transcripts in osteogenic media [36]. In contrast, the principal aim of this work was to investigate the feasibility of loading the B-HA scaffolds with unprocessed BM aspirates, as happens in the operating theatre, and to study the attachment, survival and gene expression of BMSCs on B-HA in comparison to that of cMSCs. We also used standard, sintered, isomorphic, highly porous S-HA scaffolds as control and standard SM media that are established for growing fresh BMSCs from unprocessed BM aspirate. 

We tested cMSCs and BMSCs from a broad age range of donors (19–65 years old) and provided a gene expression dataset for a large number of genes reflecting MSC differentiation and trophic capacities and angiogenesis-related genes. We observed a clear distinction between the levels of expression of genes between BMSCs and cMSCs but not so much between B-HA and S-HA. This indicated that, in terms of MSC gene expression maintenance, B-HA was not inferior to S-HA. The differences between BMSCs and cMSCs present within B-HA (or S-HA) resembled those found between uncultured and 2D-cultured MSCs, as previously shown by us and others [22,25]. These differences primarily relate to the better maintenance of adipogenic differentiation capacity in uncultured MSCs. Overall, our gene expression findings revealed good maintenance of undifferentiated MSC transcriptional signature with prominent osteogenic and adipogenic gene expression profiles up to 4 weeks in standard, static MSC culture conditions.

Secondly, we successfully showed that B-HA supported the attachment of BMSCs to the same level as S-HA (nearly all colony-forming MSCs were attached). The survival of MSCs in both scaffolds followed a similar pattern of an initial decline at week 1 followed by a ‘recovery stage’ at week 2 and, again, a decline at week 4. These patterns were not specific to B-HA, which argues against a specific inhibitory environment provided by B-HA, and there remains a possibility that some cells remained attached to scaffolds after our collagenase/trypsin-based extraction treatment. This could also be attributed to the unique porosity of B-HA scaffolds that might have prevented the removal of all cells from the pores in the first week. Future work should utilise in situ confocal staining for cell nuclei or cell metabolic activity or DNA assays to better quantify the maintenance of MSCs on these scaffolds. We also noted that BMSC maintenance on both scaffolds was lower than cMSC maintenance, possibly due to the extreme rarity of MSCs in BMA and, subsequently, their lower seeding density per scaffold (on average 16 and 13 BMSCs cells per B-HA and S-HA, respectively, compared to an average of 2 × 10^5^ and 1.6 × 10^5^ cMSCs, respectively). Future studies should investigate whether the pre-treatment of B-HA with a biocompatible ECM, such as collagen [15,37], could improve MSC maintenance inside the scaffold.

As mentioned, B-HA possess a unique structure suitable for blood vessel ingrowth into the construct. For the development of vascularised long-bone grafts, nature-inspired as well bioprinted scaffolds have been tested in the past. B-HA has been previously shown to have enhanced mechanical strength and bone regeneration abilities [13]. Using a 3D printing, Sprio et al. have demonstrated good colonisation of Haversian-canal-mimicking pores with both MSCs and HUVECs, as well as the clear benefits of MSC/HUVEC co-culture for both osteogenesis and angiogenesis, compared to MSC- or HUVEC-alone groups [29]. In our study, we confirmed the benefits of co-culturing cMSCs and HUVECs, at least in terms of VEGF production. In contrast to Sprio et al.’s 2020 study [13], we did not use a sequential seeding strategy or grown cells in the osteogenic media. The optimal way of establishing vascularised bone models, in terms of cell seeding, culture conditions and media used, remains to be established and validated between the different groups [38]. To capitalise on the unique water transport ability of the wood templates, cell seeding could be performed using a ‘bottom-up’ approach, as recently demonstrated in Liu et al.’s study [39]. Nevertheless, despite some differences in the methodologies and MSC types used, our results are consistent with those shown by Simone et al. [13], in that the B-HA scaffolds provide a beneficial environment for angiogenesis, co-existence of MSCs and endothelial cells and the production of VEGF.

## 5. Conclusions, Limitations and Future Perspectives

For the first time, we have performed a study comparing cMSCs and BMSCs on B-HA scaffolds using a wide panel of genes. We provide evidence for B-HA supporting the attachment and survival of cMSCs and BMSCs for up to 4 weeks as indicated by gene expression data. We also found higher levels of VEGF in cMSCs co-cultured with HUVECs on day 14. The data were found to be encouraging towards future studies involving a larger donor cohort of human samples.

This study is limited by the number of donors (*n* = 7 for cMSCs and *n* = 6 for BMSCs) for experiments with scaffolds; however, similar studies with B-HA or S-HA had utilised even fewer cultures or single immortalised cell lines [12]. We acknowledge the variation and the low abundance of MSCs in BMA, which restricted our flow cytometry and VEGF quantification experiments only to cMSCs [40]. We also acknowledge the donor variations that led to the lack of statistical significance in gene expression levels despite interesting trends being observed. 

This further emphasises the importance of studying primary human cells rather than cell lines from mouse models, particularly from the individuals with co-morbidities [41,42] in whom long-bone defects are particularly difficult to heal. Future work involving a higher number of donors in each set of experiments and introducing imaging techniques will provide further insight into the ability of B-HA scaffolds to encourage cellular activities, including angiogenesis support and blood vessel ingrowth. This will help to shed light on the potential of B-HA scaffolds in providing the ideal base for bone regeneration after fracture. ELISA experiments following loading the B-HA with fresh BM aspirates will provide further essential data for the use of these scaffolds in bone regeneration applications. Furthermore, future in vivo studies in large-animal, long-bone defect models [30,43] would be eventually required for the translation of these scaffolds to humans.

Overall, we successfully demonstrated that the B-HA scaffolds support the attachment and survival of human cMSCs and BMSCs. We showed higher VEGF release from supernatants collected with cMSC + E co-cultures, which when put together with complementary data from others, is encouraging for future applications of biotemplated scaffolds for large-bone defect repair.

## Data Availability

The data used to support the findings of this study are available from the corresponding author upon reasonable request.

## Supplementary Materials

The following are available online at https://www.mdpi.com/article/10.3390/bioengineering9010001/s1, Figure S1: Microscopic images of both scaffolds under transmitted light at 4X, Table S1: List for used genes, Table S2: VEGF release data from *n* = 3 BMSC donors.
