# Supplementary material for: Evaluation of Human Bone Marrow Mesenchymal Stromal Cell (MSC) Functions on a Biomorphic Rattan-Wood-Derived Scaffold: A Comparison between Cultured and Uncultured MSCs
Source: Bioengineering (Basel). 2021 Dec 21;9(1):1. doi: 10.3390/bioengineering9010001 (PMC8773042; doi:10.3390/bioengineering9010001)
Supplement: Supplementary file 1 [file bioengineering-09-00001-s001.zip › bioengineering-1462535-supplementary.pdf]

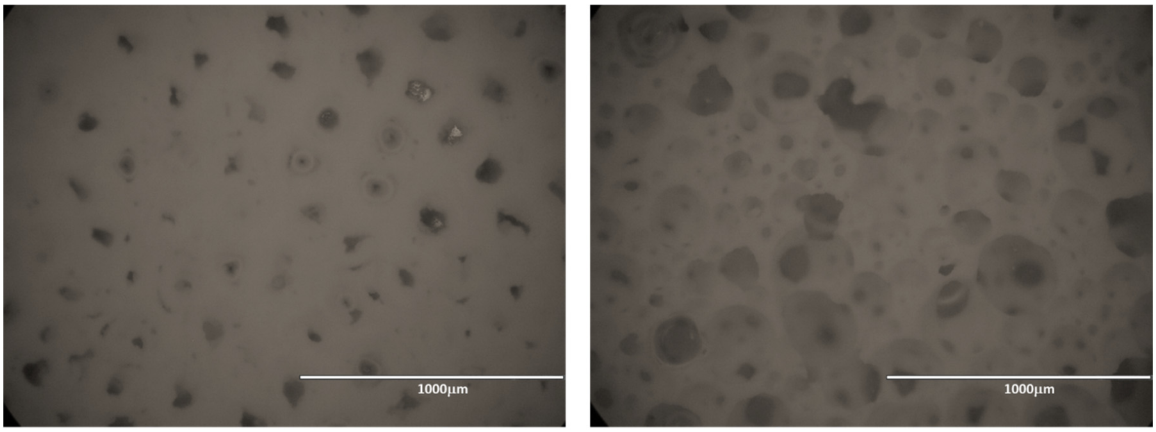

Supplementary Figure S1. Microscopic images of both scaffolds under transmitted light at 4X.

Supplementary Table S1. List for used genes.

Sr.no	Gene Abbreviation	Function
1	*RUNX2*	osteogenesis and bone remodeling
2	*ALP*	
3	*IBSP*	
4	*COL1A1*	
5	*BGLAP*	
6	*SPP1/OPN*	
7	*SPARC*	
8	*RANKL/TNFSF11*	
9	*OPG/ TNFSF11B*	
10	*ANKH*	
11	*GREM1*	
12	*E11/PDPN*	
13	*BMP2*	
14	*OMD*	
15	*PTHLH*	Chondrogenesis and cartilage damage
16	*SOX9*	
17	*COMP*	
18	*ACAN*	
19	*MMP1*	
20	*MMP2*	
21	*MMP3*	
22	*MMP13*	
23	*MMP9*	
24	*ADAMTS4*	
25	*COL10A1*	
26	*SERPINE1*	
27	*STMN2*	
28	*TIMP1*	

29	*TIMP2*	
30	*TIMP3*	
31	*PTGS2*	
32	*PPAR-γ*	Adipogenesis
33	*FABP4*	
34	*CXCL12*	Cellular interactions
35	*VEGFA*	growth factors/receptors
~~36~~	*Thy1 (CD90)*	
37	*VEGFC*	
38	*(CD73)/NT5E*	
39	*PDGFRB*	
40	*EGFR*	
41	*TGFBR2*	
42	*FGFR1*	
43	*FGFR2*	
44	*PTPRC (CD45)*	
45	*P21*	senescence and cell cycle
46	*P53*	
47	*P16*	
48	*HPRT1*	Control

Supplementary Table S2. VEGF release data from *n* = 3 BMSC donors.

**Age**	**Gender**	**VEGF levels (pg/mL)**
52	Female	146.97
45	Male	148.73
41	Female	99.48
